# Adsorption of malachite green from aqueous solutions using a novel SnO_2_/PANI-Co-PPy nanocomposite

**DOI:** 10.1039/d6ra01276k

**Published:** 2026-05-08

**Authors:** Gourang Damle, Alok Tiwari, Shivendu Saxena, Vishal Sandhwar, Diksha Saxena, Vishal Mishra, Dipak Jadhav

**Affiliations:** a Department of Chemical Engineering, Parul Institute of Technology, Parul University Vadodara-391760 India alok.tiwari30232@paruluniversity.ac.in; b School of Biochemical Engineering, IIT (BHU) Varanasi-221005 India; c School of Civil and Environmental Sciences, JSPM University Pune Maharashtra 412207 India

## Abstract

Water pollution from dye-containing industrial effluents poses significant environmental and health threats, particularly due to persistent cationic dyes like malachite green (MG). The present work demonstrates a novel modification of a green process for synthesizing tin oxide (SnO_2_) by copolymerising polyaniline (PANI) and polypyrrole (PPy) *via in situ* polymerisation for the removal of MG dye from aqueous solutions. The SnO_2_/PANI-Co-PPy nanocomposite was analyzed analytically using Fourier transform infrared (FTIR) spectroscopy, X-ray diffraction (XRD), scanning electron microscopy-energy-dispersive X-ray spectroscopy (SEM-EDX), and Barrett–Joyner–Halenda (BJH) and Brunauer–Emmett–Teller (BET) analyses. The result showed that the uniform layering of PANI and PPy on the SnO_2_ surface enables tight bonding between the polymers and SnO_2_. Several factors influence the adsorption of MG dye from synthetic simulated wastewater by SnO_2_/PANI-Co-PPy, including adsorbent dose (5 to 20 mg), time (15 to 60 minutes), pH (4 to 11), and temperature (303 to 343 K). The surface area available for the adsorption was 20 m^2^ g^−1^ with a pore size of 13.09 nm, showing maximum adsorption capacity (*Q*_m_) of 1250 mg g^−1^ and 97.06% removal at an optimized dose of 12.5 mg L^−1^, pH 9 and initial MG concentration of 50 mg L^−1^ in 30 minutes. The kinetic study of the SnO_2_/PANI-Co-PPy nanocomposite showed the suitability of the second-order kinetic model, achieving equilibrium in 30 minutes at an initial concentration of 50 mg L^−1^. For the composite, Δ*S* was >0 (high randomness at the solid–liquid interface) along with exothermic characteristics (Δ*H* < 0). Overall, SnO_2_/PANI-Co-PPy was an effective adsorbing material with high removal efficiency for treating wastewater contaminated with MG dye.

## Abstract 2

Water pollution from dye-containing industrial effluents poses significant environmental and health threats, particularly due to persistent cationic dyes like malachite green (MG).

## Graphical abstract

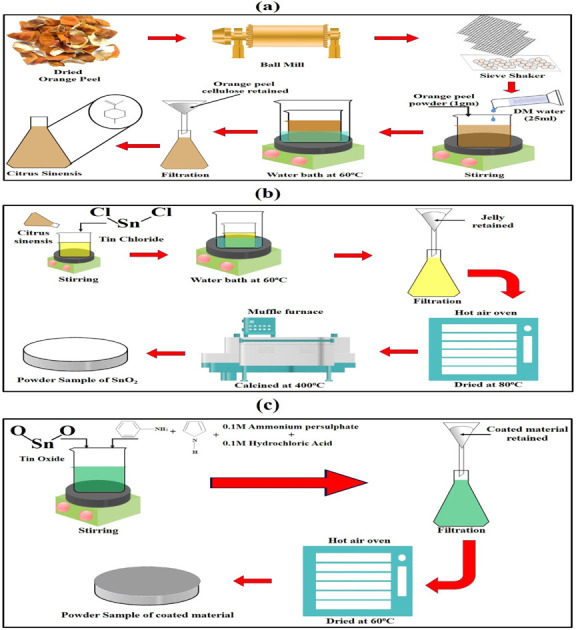

## 1. Introduction

Urbanization and industrialization have led to the discharge of large volumes of untreated water into the environment, posing a critical threat to both human health and aquatic life. According to the Central Pollution Control Board (CPCB-August 2020), urban areas generate 72 368 MLD. The installed sewage treatment capacity is 31 481 MLD, while the operation capacity is 26 869 MLD. So, only 28% is getting treated, while 78% is discharged directly to rivers, lakes and aquifers.^1^ Industries such as textiles, paper, leather tanning, food processing and cosmeticscontribute significantly to water usage, resulting in high volumes of wastewater that often overwhelm treatment capabilities.^2,3^

Dye manufacturing, particularly cationic dye production, poses environmental challenges due to the toxic contaminants released into wastewater. Dyes typically contain harmful compounds, including heavy metals and organic pollutants, which jeopardize ecological health.^4,5^ Cationic dyes, characterized by their increased water solubility and toxicity, represent a significant portion of dye concentrations in effluents. It has been estimated that approximately 280 000 tons of textile dyes are discharged annually worldwide.^6^ Notably, the dyes such as methylene blue (MB), malachite green (MG), and rhodamine B (RB) have been shown to adversely affect soil fertility and crop yield and pose human health risks.^5^

Studies on effluent treatment methodologies has spurred interest in various conventional and innovative technologies. Traditional methods, such as photocatalysis and electrochemical treatments, have been explored; however, these often face challenges related to cost, sustainability, and operational complexity.^7,8^ Adsorption has emerged as a particularly promising method owing to its simplicity and environmental friendliness; it effectively utilizes materials with high surface areas like activated carbon and biochar.^9^

The research on effluent treatment methods has sparked a lot of interest in advanced oxidation processes and separation technologies. Methods such as photocatalysis and electrochemical treatments have shown the capacity for dye remediation, but their industrial application is limited by energy-intensive and complex technologies with high operation costs. On the other hand, adsorption has emerged as an excellent alternative due to its cost-effectiveness, operational simplicity, and environmental sustainability.^10–15^

Metal oxide-based adsorbents, particularly tin oxide (SnO_2_), have shown significant potential for treating wastewater contaminated with MG dye, prompting extensive research into hybrid composites of SnO_2_ with conductive polymers such as polyaniline (PANI) and polypyrrole (PPy).^16–18^ This hybridization aims to enhance adsorption properties, leveraging the redox activity and biocompatibility of these materials for effective water treatment.^5,8^ Literature review indicates the effectiveness of PANI/SnO_2_ and PPy/SnO_2_ in dye remediation, demonstrating potential pathways for developing composite adsorbents.^16–18^ Though binary systems have been explored, comprehensive studies investigating the efficacy of PANI and PPy simultaneously co-polymerized with SnO_2_ for cationic dye absorption are restricted and currently lacking, indicating a significant area for exploration.^19,20^ The co-polymerized PANI and PPy provided a higher surface area than either PANI or PPy alone. It also overcame the issue of resistance to π-interactions.^21^ The other advantages of this combination are easy bulk synthesis, low cost, non-toxicity and biocompatibility.^19,20^

This study aims to synthesize a novel SnO_2_/PANI-Co-PPy nanocomposite, examining its potential for adsorbing MG dye from aqueous solutions. The impact of key factors, such as pH, dosage, contact time, and temperature, on MG adsorption was evaluated. In order to elucidate the adsorption characteristics and performance of this approach for escalating environmental concerns, a comprehensive evaluation of adsorption kinetics, isotherm modelling, and thermodynamics at the solid–liquid interface was conducted. SnO_2_ incorporated into the copolymer matrix prevents the leaching of nanoparticles, thus preventing the contamination of wastewater and reducing carbon footprint.^17,18^

## 2. Materials and methods

### 2.1 Materials

Tin chloride (SnCl_2_) of analytical grade and orange peels were used to prepare the tin oxide nanoparticles. Aniline (C_6_H_7_N) and pyrrole (C_4_H_5_N) of 99% purity, analytical grade ammonium persulfate ((NH_4_)_2_S_2_O_8_) and hydrochloric acid (HCl) were used to synthesize SnO_2_/PANI-Co-PPy. MG dye (97%) was used to prepare a 1000 ppm stock solution. Sodium hydroxide (NaOH) and HCl with a purity of 99% were utilized to maintain the initial pH of the dye solution. All the chemicals were purchased from Sigma-Aldrich, USA.

### 2.2 Material synthesis

The synthesis of SnO_2_/PANI-Co-PPy is a 2-step process. Initially, *Citrus sinensis* is extracted from the orange peel, which is then further used in the synthesis of SnO_2_. SnCl_2_ was added as a precursor in *Citrus sinensis* to synthesize SnO_2_ by the sol–gel method.^22^ In the second stage, *in situ* polymerization was adopted for coating with the conductive polymer (PANI and PPy).

#### 2.2.1 Extraction of *Citrus sinensis* from orange peel crush

**Fig. 1:**
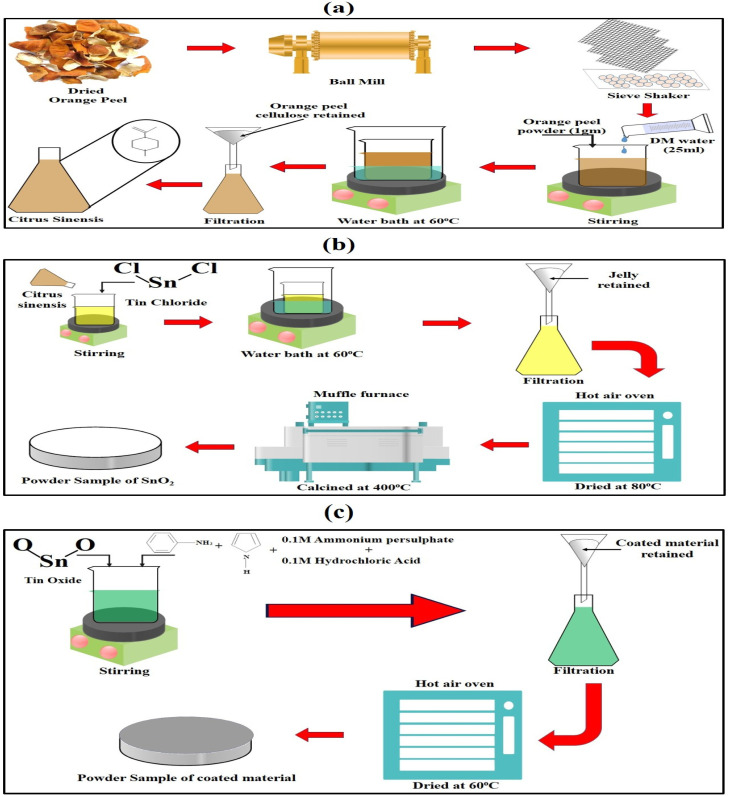
(a) *Citrus Sinensis* extract preparation. (b) Green synthesis of SnO_2_. (c) *In situ* polymerization.

Orange peels were procured from a fruit shop situated in the university campus. The peels were washed multiple times with distilled water (DW) to remove dirt and contamination. Subsequently, the peels were sun-dried for 5 days. Following this initial drying process, the sun-dried peels were placed in the oven at 50 °C for 5 hours for further dehydration. Furthermore, the dried peels were crushed into powder using a ball mill. Subsequently, a sieve shaker was used to separate the powdered materials into fractions of different sizes. The particles with a size less than 200 microns were used for further processing of the extraction of *Citrus sinensis*.^22^ The extraction process involved mixing fine orange peel powder with DW in a 1 : 25 (w/v) ratio. The mixture was then stirred at 1000–1300 rpm in a magnetic stirrer for 2 h. This is followed by heating the mixture at 60 °C for 1 h. The mixture was subsequently allowed to rest for 30 minutes, during which solid particles settled to the bottom. The resultant solution was then filtered with Whatman filter paper (Grade 1) to obtain the *Citrus sinensis* extract.^22^ The schematic representation of the entire process is illustrated in Fig. 1(a).

#### 2.2.2 SnO_2_ NPs from *Citrus sinensis*

In the next phase, SnCl_2_ was mixed with the *Citrus sinensis* extract in a ratio of 2 : 42.5 (w/v). The mixture was stirred at 1000–300 rpm using a magnetic stirrer for one hour, resulting in a pale-yellow colouration, indicative of the conversion of tin chloride to tin oxide. This mixture was thereafter maintained in a water bath at a temperature of 60 °C for 7 hours, facilitating the formation of a plasticised jelly.^22^ This jelly was subsequently filtered using filter paper (grade 1) and washed with DW^23,24^ and isopropyl alcohol (IPA)^25^ to achieve a neutral pH. Thereafter, the jelly was dried in a hot-air oven at 80 °C for 24 hours to remove residual IPA and DW. The dried jelly underwent calcination in a muffle furnace at 400 °C for one hour, which activated and increased the crystallinity of the nanoparticles and yielded an off-white powder. The schematic of the entire process is illustrated in Fig. 1(b).

#### 2.2.3 *In situ* polymerization to synthesize SnO_2_/PANI-Co-PPy

To synthesize the polymer composite, 1 g of SnO_2_ NPs was added to 250 mL of a 0.1 M HCl solution (to break the bonds of aniline and pyrrole). Additionally, 0.75 g of aniline and 0.75 g of pyrrole were added. The mixture was stirred for one hour at 900–1200 rpm at 0 °C–5 °C in an ice bath. Following this mixing period, 50 mL of a 0.1 M ammonium persulfate solution was added dropwise as a polymerization initiator, resulting in a greenish solution.^26^ This mixture was continuously stirred for six hours at a temperature of 0 °C–5 °C. Subsequently, it was allowed to stand undisturbed in a dark environment to enhance bonding.^27^ The material was then washed with DW and IPA. The filtered material was then dried in a hot air oven at 60 °C for 24 hours. The schematic representation of the process is demonstrated in Fig. 1(c).

### 2.3 Analytical method

For FTIR spectroscopy, Alpha II, Bruker Optics, USA (Massachusetts) was used, and spectra were recorded from 4000 cm^−1^ to 400 cm^−1^ to identify the characteristics of functional groups and chemical bonding in the synthesized particles. The SU3800 Hi-SEM model (Hitachi High-Tech India Pvt. Ltd, Japan) was utilized to analyze the morphology and topography of the prepared sample. Bruker Analytical X-ray Solutions (AXS), USA, was used for the XRD analysis. Patterns were recorded using a D6 Phaser diffractometer equipped with a 1.2 kW X-ray tube source. The data, collected at a precision of 0.01°, were used to determine the crystallinity, lattice planes, and crystallite size. A decrease in peak intensity and broadening upon polymer coating suggested structural modification of SnO_2_. Specific surface area was studied by utilizing a BET analyzer, and nitrogen adsorption–desorption isotherms were estimated at −196 °C (77 K) using the BELSORP-max II system (MicrotracBEL Corporation, Japan). The pore size distribution was analyzed with the BJH approach. This analysis provided insights into the porosity and surface properties of the composite material.

### 2.4 Treatment method

The efficiency of the SnO_2_/PANI-Co-PPy nanocomposite was determined *via* batch adsorption of MG. Initially, a stock solution of 1000 ppm (0.25 g dye + 250 mL DI water) was prepared, and for the study, a 50 ppm sample was prepared by successive dilution. In order to study the influencing parameters, concentration, time and volume were fixed at 50 ppm, 30 minutes, and 100 mL, respectively. The removal efficiency and adsorption capacity were calculated by using eqn (1) and (2) (ref. 20 and 25) for the various affecting parameters, such as SnO_2_/PANI-Co-PPy dosage, initial pH, time and temperature as follows:1
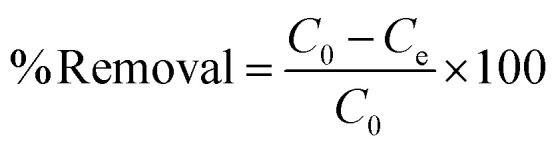
2
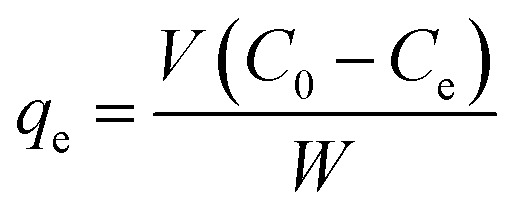


To evaluate the dosage effect, the dose was varied from 5 mg to 20 mg, without varying the other parameters like initial pH and temperature. To validate the optimized dose time, a study was carried out for 60 minutes, with samples collected every 15 minutes. The initial pH study was carried out at the optimized dose of 12.5 mg L^−1^ with previously mentioned fixed parameters in the range 4 to 11. The initial pH was adjusted by using 0.1 M NaOH and 0.1 M HCl. To validate the optimized pH, the point of zero charge was calculated using the pH drift method. For the point of zero charge calculation, samples with initial pH varying from 4 to 11 were prepared with other fixed parameters and agitated for 48 hours. The final pH of the solutions was measured and plotted against the initial pH. The point at which the final pH = initial pH (passing through the origin) was considered as pH_pzc_. The effect of temperature was evaluated by adjusting all optimized parameters from 303–343 K. For the evaluation of adsorption isotherms, kinetic study, and thermodynamic terms, a batch study was carried out at the optimized values of process parameters (dose = 12.5 mg L^−1^, initial pH = 9, and temperature = 303 K). Langmuir, Freundlich, and Temkin adsorption isotherms were used in the study, in which only the concentration was varied from 10 to 50 ppm. The kinetic study was carried out at a 50 ppm concentration for 60 minutes. A thermodynamic study of adsorption was carried out from 303 to 343 K at an initial concentration of 50 ppm.

## 3. Results and discussion

### 3.1 Characterization of synthesized nanoparticles

#### 3.1.1 FTIR

**Fig. 2:**
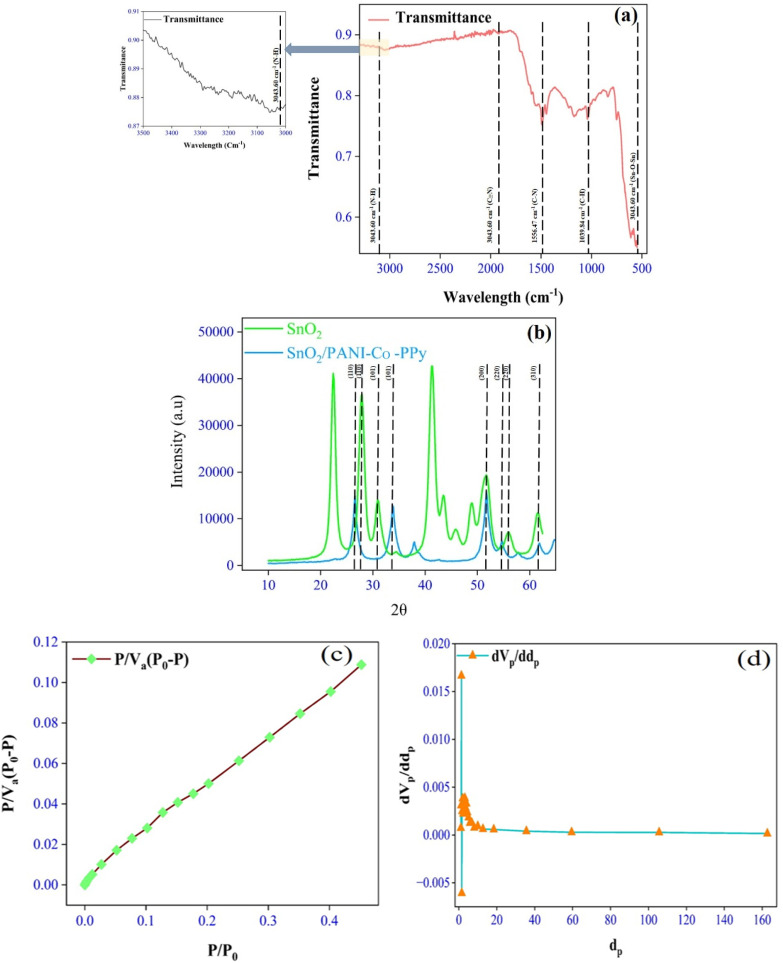
(a) FTIR spectrum of SnO_2_/PANI-Co-PPy. (b) XRD patterns of SnO_2_ and SnO_2_/PANI-Co-PPy. (c) BET curve of SnO_2_/PANI-Co-PPy. (d) BJH curve of SnO_2_/PANI-Co-PPy.

The molecular bonding of the modified SnO_2_ was examined by FTIR analysis in the wavenumber range 400–4000 cm^−1^, as demonstrated in Fig. 2(a). The prominent absorption band at 561.79 cm^−1^ confirmed the presence of Sn–O–Sn in the synthesized material.^28^ In addition to this, an overlapping band at 609.94 cm^−1^ on the Sn–O–Sn peak affirmed the modification with the co-polymer.^29^ There is a hydrogen bond between SnO_2_ and the copolymer as a characteristic peak observed at 3043.60 cm^−1^, corresponding to the stretching between N–H and O–H. Furthermore, the bands observed over the wavenumber ranges 2100–700 cm^−1^ provided evidence of successful surface modification. The bands at 2191.28 cm^−1^, 2164.27 cm^−1^, and 1924.13 cm^−1^ were assigned to the C≡N stretching, indicating copolymer incorporation.^29,30^ Also, the peaks observed at 1556.47 cm^−1^ and 1493.62 cm^−1^ validated co-polymerization, as the C–N stretching and C–C bonding of the benzoid rings were observed.^30^ In additions, peaks corroborating the co-polymerization are 1171.55 cm^−1^ (C–O vibrations), 1039.84 cm^−1^ (C–H bending),^30^ 837.13 cm^−1^ (aromatic C–H bond vibration),^27^ and 751.13 cm^−1^ (out-of-plane bending of C–Cl).^30^ These results indicate that SnO_2_/PANI-Co-PPy with effective surface functionalization and bonding interactions was effectively synthesized.

#### 3.1.2 XRD

The XRD spectra of unmodified and modified SnO_2_ are shown in Fig. 2(b). For the unmodified SnO_2_, sharp peaks were observed at 2*θ* values 27.94°, 31.08°, 51.56°, 55.9°, and 61.43°, which corresponded to the (110), (101), (211), (220), and (310) crystalline planes, respectively. The results were similar to the standard tetragonal rutile SnO_2_ structure.^31^ On the other hand, in modified SnO_2,_ similar peaks appeared at 26.59°, 33.92°, 51.71°, 54.70°, and 61.88° corresponding to the same crystalline planes, (110), (101), (211), (220), and (310), respectively. This peak shows reduced intensity and peak enlargement due to the amorphous nature of the conductive polymer,^32^ as shown in Fig. 2(b). The average crystallite size of the nanoparticles was calculated by the Debye–Scherrer eqn (3) as follows:3
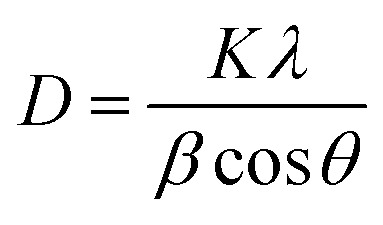
where *D* = mean crystallize size, *K* = Debye–Scherrer's constant (0.9), *λ* = wavelength used in XRD (1.54 A°), *β* = full width at half-maximum (FWHM), and *θ* = Bragg's diffraction angle in radians. The crystal sizes pre- and post-modification were 5.1943 nm and 4.6031 nm, respectively. These small sizes suggested that particles were agglomerated, and the coating of the conductive polymer did not affect the crystallization behaviour.^29^

#### 3.1.3 BET and BJH

The area and pore size provided by the adsorbent play a significant role in adsorption.^33^ BET and BJH are shown in Fig. 2(c and d). Table 1 displays the surface area, mean pore volume, and average pore diameter. According to the International Union of Pure and Applied Chemistry (IUPAC) standards, the SnO_2_/PANI-Co-PPy nanocomposite was classified as mesoporous, as the average pore diameter was 9.3428 nm. The SnO_2_/PANI-Co-PPy nanocomposite also displayed a surface area of 20.43 m^2^ g^−1^ and a mean pore volume of 6.691 × 10^−2^ cm^3^ g^−1^. As the molecular size of MG dye is 0.82 nm,^33^ it can easily reach the active site, which makes SnO_2_/PANI-Co-PPy a potential adsorbent for dye removal. The composite has a higher surface area and pore volume compared to the values reported by Tabassum N. *et al.* and Umeh C. T. *et al*.^33,34^

**Table 1:** BET and BJH parameters

Parameters	Values
Surface area (m^2^ g^−1^)	20.43
Mean pore volume (cm^3^ g^−1^)	6.691 × 10^−2^
Average pore diameter (nm)	9.3428

#### 3.1.4 SEM-EDX and elemental mapping

**Fig. 3:**
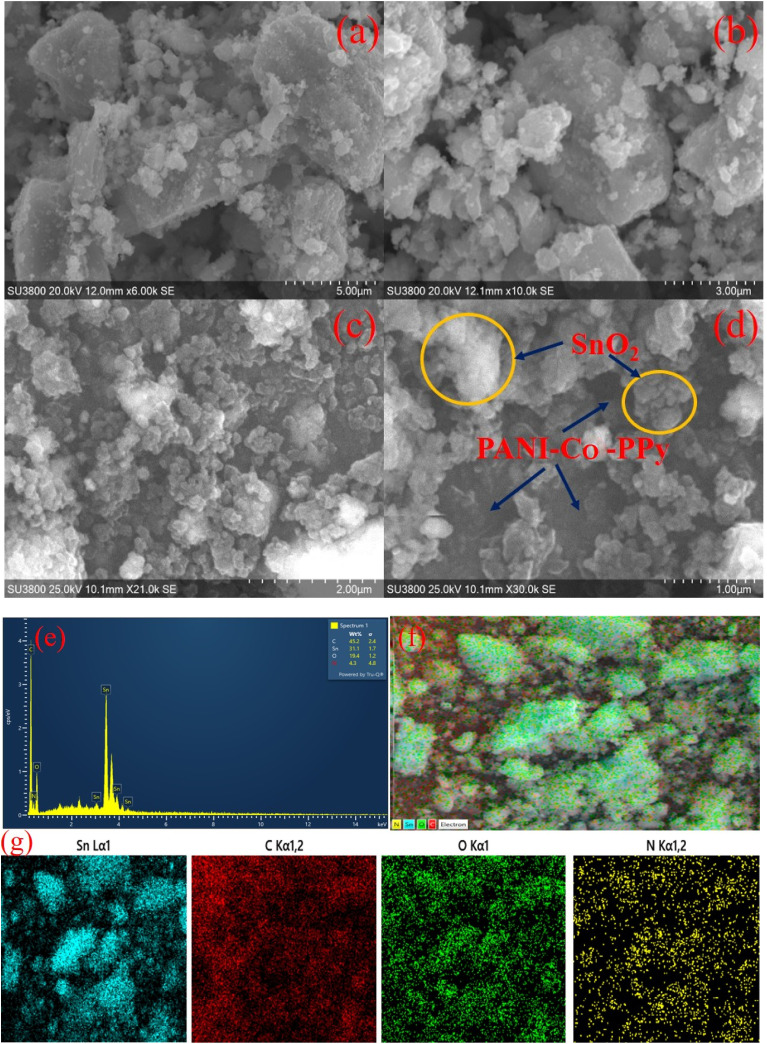
SEM images of (a and b) SnO_2_ NPs and (c and d) SnO_2_/PANI-Co-PPy. (e) EDX spectrum of SnO_2_/PANI-Co-PPy and (f and g) elemental mapping images of SnO_2_/PANI-Co-PPy.

SEM analysis was performed to examine the surface morphology of pristine SnO_2_ and SnO_2_/PANI-Co-PPy, as illustrated in Fig. 3(a–d). Fig. 3(a and b) revealed spherical clusters of SnO_2_.^22,35^ However, due to rapid precipitation during the process, agglomerated coarser particles were observed.^33^ In contrast, Fig. 3(c and d) presents a uniform distribution of PANI-Co-PPy over the SnO_2_ surface as seen from the elemental mapping results (Fig. 3(f)). However, few particles showed clear boundaries, indicating encapsulation with a copolymerized substance, along with surface functionalization.^36^ Conversely, the particles having indistinct boundaries were bonded by intimate contact with the surrounding copolymer chains.^32^ These observations affirmed the incorporation of SnO_2_ into the copolymer matrix, with the formation of a uniform and interconnected composite structure. Fig. 3(e) shows the EDX spectrum of SnO_2_/PANI-Co-PPy. This result showed the weight% of tin (Sn), carbon (C), oxygen (O), and nitrogen (N) as 31.1, 45.2, 19.4, and 4.3, respectively. The synthesized material contained only Sn, C, O, and N, which showed the high purity of SnO_2_/PANI-Co-PPy synthesized in this work.^25^ The average sample size was recorded as 0.732 nm for pristine SnO_2_ and 0.602 nm for SnO_2_/PANI-Co-PPy.

### 3.2 Dye removal studies of SnO_2_/PANI-Co-PPy

#### 3.2.1 Influence of SnO_2_/PANI-Co-PPy dosage

**Fig. 4:**
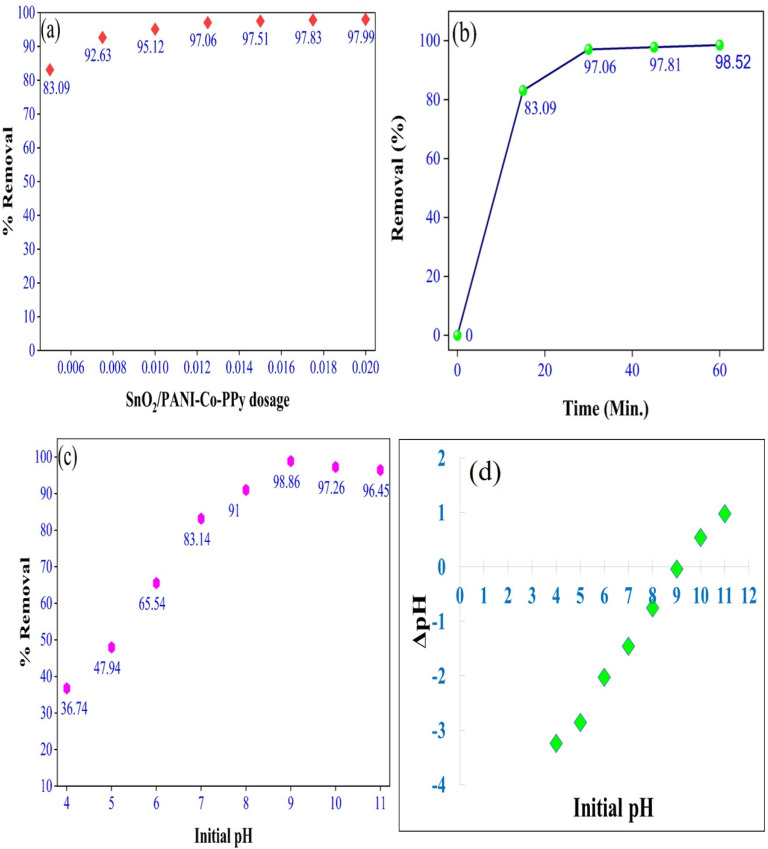
(a) Influence of the SnO_2_/PANI-Co-PPy dosage. (b) Influence of the contact time. Variance of (c) pH and (d) point of zero charge.

The effect of SnO_2_/PANI-Co-PPy dose on the removal of MG dye was estimated by varying the SnO_2_/PANI-Co-PPy doses from 5 to 20 mg L^−1^ with the initial dye concentration of 50 ppm for 60 minutes (Fig. 4(a)). Removal increased gradually with the increase in dosage up to 12.5 mg L^−1^, and 97.06% removal was achieved. However, beyond this dosage, there was less impact on the removal, indicating attainment of equilibrium.^20,37^ Another reason behind these results was the aggregation of particles with the increased dosage, leading to a reduction in the number of active sites.^38^

#### 3.2.2 Influence of contact time

The process was run for 60 minutes, and the removal was examined every 15 minutes. The study was conducted at a dosage of 12.5 mg L^−1^ SnO_2_/PANI-Co-PPy with an initial concentration of 50 ppm. Fig. 4(b) indicates that after 30 minutes of contact with SnO_2_/PANI-Co-PPy, the removal obtained is 97.06%. The root cause of this was the saturation of active sites after equilibrium,^28^ along with resistance against the diffusion of aggregated dye molecules by the SnO_2_/PANI-Co-PPy surface.^39^

#### 3.2.3 Variance of pH

The initial pH optimization was studied over the range 4 to 11. This adjustment to the desired value was made using 0.1 M HCl and NaOH.

At acidic pH (4 to 6), with a high concentration of H^+^ ions, the dye molecules were repelled by the SnO_2_/PANI-Co-PPy surface.^40^ With the increase in pH (8 to 11), the OH^−^ ion concentration increased, which attracted the dye molecule towards the surface of SnO_2_/PANI-Co-PPy^28^ (Fig. 4(c)).

The pH_pzc_ for SnO_2_/PANI-Co-PPy was observed as 9 (Fig. 4(d)). Maintaining the solution pH above pH_pzc_ imposed a negative charge on the SnO_2_/PANI-Co-PPy surface, which facilitated the adsorption of dye molecules. Below pH_pzc,_ the positive charge was present on the SnO_2_/PANI-Co-PPy surface, which repelled the dye molecules.^41,42^

#### 3.2.4 Literature comparison

The effectiveness of the synthesized SnO_2_/PANI-Co-PPy composite was compared with recently described composites for the extraction of Malachite Green (MG) dye using metal oxide-based materials (see Table 2). The literature provides clear examples of composites that exhibit good performance; however, many of them are constrained by numerous limitations or are limited by the kinetics of removal. For example, Yadav *et al.*^54^ reported a removal efficiency of 71–79% at a pH of 7–10 but required much higher dosages and longer contact times to achieve a low initial concentration of 10 ppm of MG. Similarly, removal by the TiO_2_-GO composite developed by Verma *et al.*^53^ at an initial concentration of 10 ppm was 84%, and this technology also has unresolved scalability issues as dye load increases. The analysis by Kamble *et al.*^50^ has shown that cobalt-doped TiO_2_ can be applied at higher concentrations (50 ppm); however, the time periods required to obtain equilibration are much longer than those observed in the current study. The SnO_2_/PANI-Co-PPy composite shows an exceptional combination of high-capacity adsorption and fast reaction kinetics compared to other composites. It can achieve a higher total degradation percentage than the comparative benchmarks at higher initial concentrations, with less material usage and a shorter contact time. This is a result of the synergistic relationship between the SnO_2_ nanoparticles and the conductive polymer matrix (PANI-Co-PPy), which together provide a large surface area and many active sites for a faster dye adsorption.

**Table 2:** Comparison of literature studies for the removal of MG dye

Sr. no.	Metal oxide-based nanocomposites	Degradation process	pH	Dosage (mg)	Time (min)	Temperature (K)	Initial concentration (ppm)	% Removal	Ref.
1	Ag/AgCl and Ag/AgCl-GO	Photocatalytic	—	60	20	—	15	88	47
2	MoS_2_/Mg(OH)_2_/BiVO_4_	Photocatalytic	—	80	60	—	20	41–74	48
3	CuO-Gd_2_Ti_2_O_7_	Photocatalytic	—	2.5–10	90	—	10	86	49
4	Co-doped TiO_2_	Photocatalytic	—	5	180	—	50	31–82	50
5	GO	Photocatalytic	9	100	60	—	20	85	51
6	TiO_2_-inulin-Fe_3_O_4_	Photocatalytic	—	10	—	—	—	34–81	52
7	TiO_2_-GO	Photocatalytic	10	10	13	—	10	84	53
8	β-CD-CuO/ZnO	Photocatalytic	7–10	100	180	—	10	71–79	54
9	TiO_2_/GO	Photocatalytic	5–6	—	90	—	—	48	55
10	SnO_2_	Photocatalytic	—	—	15	—	—	24	55
11	SnO_2_/SBB	Adsorption	8	30	20	303	10	52–73	28
12	MnO_2_	Adsorption	10	100	90	—	50	90	56
13	SnO_2_/PANI-Co-PPy	Adsorption	9	12.5	30	303	50	97.06	This study

#### 3.2.5. Adsorption isotherm

**Fig. 5:**
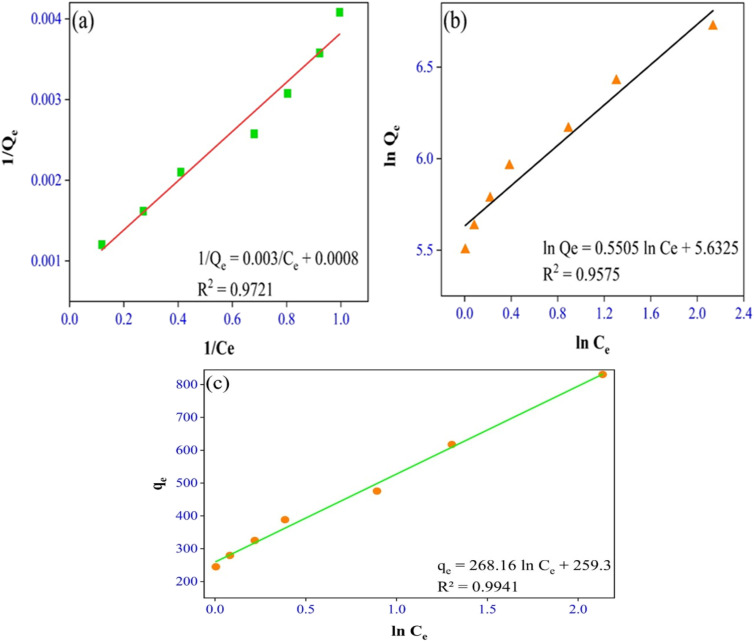
(a) Langmuir, (b) Freundlich and (c) Temkin isotherms.

In this study, data were fitted in the Langmuir, Freundlich, and Temkin isotherms, which can be seen in Fig. 5(a–c).

The following were the linearized equations of this model.^16,40^4
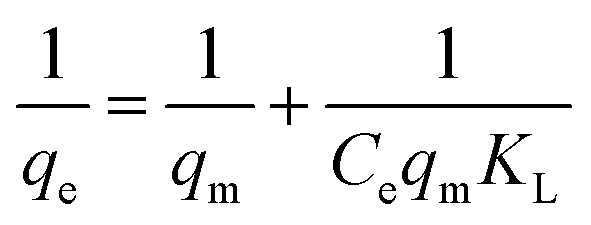
5
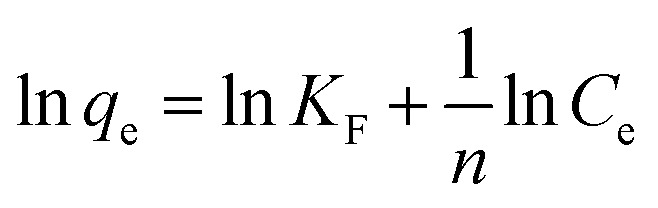

6 *q*_e_ = *B* ln *A*_T_ + *B* ln *C*_e_

where *C*_e_ = equilibrium concentration (mg L^−1^) *q*_e_ = equilibrium adsorption capacity (mg g^−1^), *q*_m_ = maximum adsorption capacity (mg g^−1^), *K*_L_ = Langmuir isotherm constant (L mg^−1^), *K*_F_ = Freundlich isotherm constant (mg g^−1^), *n* = adsorption intensity, *A*_T_ = Temkin isotherm equilibrium binding constant (L g^−1^), and *B* = Temkin isotherm constant. It is evident from the results that the Temkin isotherm fitted best into the experimental data with the *R*_2_ ≈ 1 and the separation factor (*R*_L_) value was close to 0. Also, the kinetic study favored chemisorption, reflecting the suitability of the Temkin model.

7 *R*_L_ = 1/(1 + *K*_L_*C*_0_)

The Temkin isotherm model shows that the binding energy between the molecules decreases linearly rather than logarithmically. It was confirmed that the dye molecules were adsorbed in multilayers, indicating physisorption,^37,42^ and the parameters related to the adsorption isotherms are shown in Table 3.

**Table 3:** Adsorption isotherm parameters for dye removal

Isotherms	Parameters
Langmuir	*Q*_m_ = 1250 mg g^−1^
	*K*_L_ = 0.2667 (L mg^−1^)
	*R*_L_ = 0.074
	*R* ^2^ = 0.9721
Freundlich	*n* = 1.81653
	*K*_F_ = 279.3596 (mg g^−1^)
	*R* ^2^ = 0.9575
Temkin	*B* = 268.16
	*A*_T_ = 2.6299 (L g^−1^)
	*R*_L_ = 0.0075
	*R* ^2^ = 0.9941

#### 3.2.6 Kinetic study

**Fig. 6:**
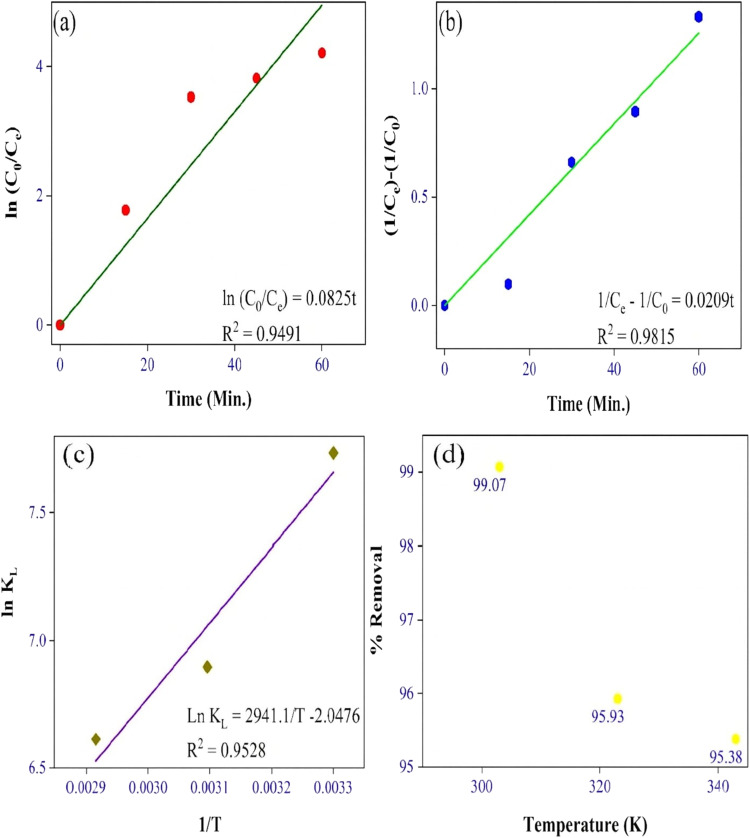
(a) First order reaction kinetics, (b) second order reaction kinetics, (c) thermodynamic study, and (d) temperature study.

The rate of this process is dependent on various factors, but surface complexity, contact time, and solute concentration are the main influencing factors.^37^ In the present work, the kinetics study is conducted using first and second-order reaction models. The mathematical models (linear fit) are represented by eqn (8) and (9) as follows:8
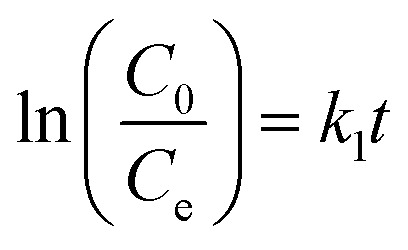
9
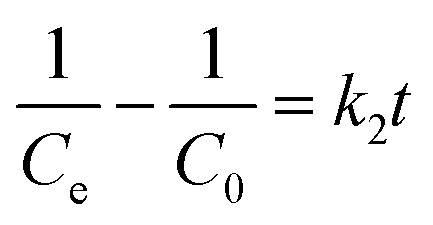
where *k*_1_ (min^−1^) and *k*_2_ (g mg^−1^ min^−1^) are the rate constants of 1st and 2nd order, respectively. *C*_0_ is the initial dye concentration (mg L^−1^), and *C*_e_ is the concentration at time *t* (mg L^−1^). The linear fitting of this order for MG dye is shown in Fig. 6(a and b). The first and second order *R*^2^ values of MG dye were 0.94 and 0.98 (see Table 4).

**Table 4:** Kinetic parameters for the MG dye removal

SnO_2_/PANI-Co-PPy dosage (g)	Order of the reaction	Rate constant	*R* ^2^
0.0125	1st	0.0825 (min^−1^)	0.9491
	2nd	0.0209 (g mg^−1^ min^−1^)	0.9815

The kinetic modelling showed that the adsorption was rate-limiting with a best fit in 2nd order (*R*^2^ value was approximately 1), and the value of the rate constant was in the range.^28,35^ Also, Tabassum N. *et al.*^33^ and Sharma P. *et al.*^28^ have reported the suitability of the second-order kinetic model in deciphering the adsorption mechanism of the MG dye.

#### 3.2.7 Thermodynamics study and influence of temperature

In the present work, thermodynamic parameters such as Gibbs' free energy (Δ*G*), enthalpy change (Δ*H*) and entropy change (Δ*S*) were calculated. For calculating the thermodynamic parameters, the following equations were used:

10 Δ*G* = −*RT* ln *k*_c_

11 Δ*G*^0^ = Δ*H*^0^ − *T*Δ*S*^0^

12
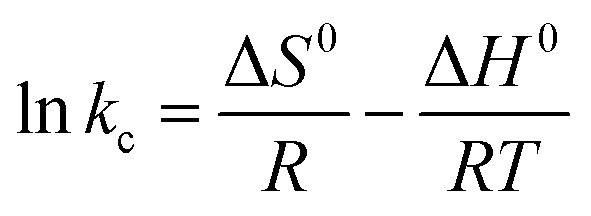
where *R* = gas constant (8.314 J mol^−1^ K^−1^), *T* = temperature (K), and *K*_c_ = equilibrium constant (L mg^−1^). From Fig. 6(c), the enthalpy and entropy change for MG dye can be determined by using the slope and intercept of eqn (10).

Negative values of Δ*G* and Δ*H* suggested that the process was spontaneous and exothermic for MG dye adsorption, which was also validated through a temperature study (Fig. 6(d)). The negative value of Δ*S* reflected less randomness at the solid–liquid interface^9^ (Table 5). These results were consistent with those of Tabassum N. *et al.* and Sharma P. *et al*.^28,33^

**Table 5:** Thermodynamic parameters for MG dye removal

SnO_2_/PANI-Co-PPy dosage (g)	Temperature (K)	Δ*G* (kJ mol^−1^)	Δ*H* (J mol^−1^)	Δ*S* (J K^−1^)
0.0125	303	−19483.9	−24452.31	−17.0237
	323	−18521		
	343	−18856.5		

#### 3.2.8 Plausible mechanism of SnO_2_/PANI-Co-PPy

**Fig. 7:**
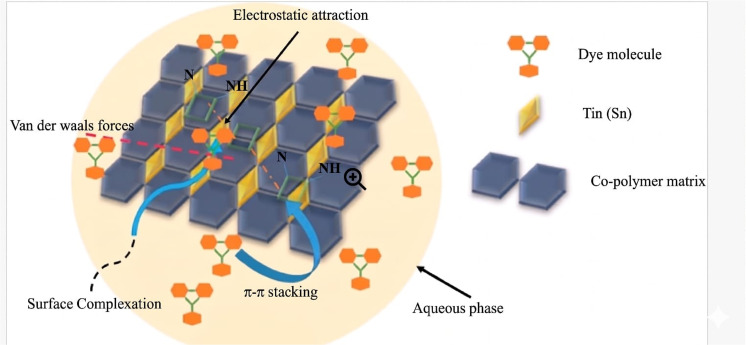
Plausible mechanism.

The higher adsorption capacity of SnO_2_/PANI-Co-PPy was due to electrostatic interaction, π–π stacking, hydrogen bonding and surface complexation (Fig. 7). As MG dye contains the aromatic ring structure and the co-polymerized conductive polymer comprises conjugated aromatic rings, these produce the strong molecular Velcro effect, which aids in the removal of dye from aqueous solutions.^43^ The presence of SnO_2_ in the composite averted the agglomeration of polymers, which in turn provided 20 m^2^ g^−1^ area with a higher number of active sites, which supported the removal of MG dye.^44^ At pH 9, the adsorbent surface was negatively charged due to OH^−^ ions. As the MG dye bears a positive charge, the process leads to higher dye removal due to electrostatic attraction.^45^ The presence of nitrogen in the conductive polymer offered a surface for hydrogen bonding with dye functional groups, which benefits in the removal.^46^

## 4. Conclusions

This study successfully demonstrated the preparation of the SnO_2_/PANI-PPy nanocomposite *via* a green process. *Citrus sinensis* extract was used as a reducing agent for the synthesis of the SnO_2_/PANI-PPy nanocomposite. The synergetic effect of green-synthesised metal oxide with a conductive polymer matrix provided a significant advancement in developing a high-throughput, eco-friendly adsorbent.

The physico-chemical characterization results demonstrated the unique mesoporous structure and specific surface area of 20 m^2^ g^−1^, which was instrumental in achieving the exceptional adsorption capacity of 1250 mg of MG g^−1^ of the SnO_2_/PANI-PPy nanocomposite. Unlike the homopolymer systems, the combined effect of the PANI-PPy co-polymerisation increases molecular interactions at the solid–liquid interface, facilitating the rapid and efficient dye removal. Kinetic and thermodynamic modelling studies were best described by the Temkin model, showing multi-layer interaction and non-uniform distribution of binding energies.

The significant finding of this study underscores the potential of SnO_2_/PANI-PPy as a robust and sustainable solution for industrial effluents containing cationic dyes. Future work will focus on the regeneration potential, application of the adsorbent in real-world applications and in the multi-component water system.

## Author contributions

Gourang Damle – conceptualization, methodology, and writing – original draft, Alok Tiwari – conceptualization, resources, investigation, and writing – review and editing, Shivendu Saxena – data curation, formal analysis, and writing – review and editing, Vishal Sandhwar-writing – review and editing and formal analysis, Diksha Saxena-writing – review and editing, Vishal Mishra – writing-review and editing, validation, and investigation, and Dipak Jadhav – writing-review and editing.

## Conflicts of interest

The authors declare that the research was conducted in the absence of any commercial or financial relationships that could be construed as a potential conflict of interest.

## Data Availability

The data can be provided upon request.
